# Two New Iridoid Glycosides from the Root Barks of *Sambucus williamsii* Hance

**DOI:** 10.3390/molecules17021830

**Published:** 2012-02-13

**Authors:** Hai-Xue Kuang, Hua Han, Bing-You Yang, Liu Yang, Hai Jiang, Qiu-Hong Wang

**Affiliations:** Key Laboratory of Chinese Materia Medica, Heilongjiang University of Chinese Medicine, Ministry of Education, Harbin 150040, China

**Keywords:** *Sambucus williamsii* Hance, root barks, iridoid glycosides

## Abstract

Two new iridoid glycosides, named williamsoside C (**1**) and williamsoside D (**2**) were isolated from the root barks of *Sambucus williamsii* Hance. Their structures were established on the basis of extensive spectroscopic analysis (1D, 2D NMR and HRESIMS) and chemical studies as α-D-glucopyranosyl (1→2)-β-D-fructofuranosyl (4→6)-β-morroniside (**1**) and 7β-*O*-ethyl morroniside-(6′-*O*-7′′)-β-morroniside (**2**), respectively.

## 1. Introduction

**Figure 1:**
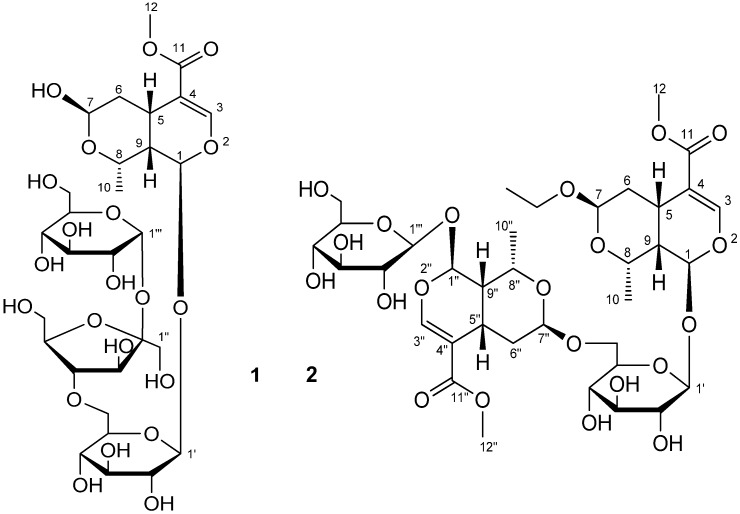
Structures of compounds **1** and **2**.

*Sambucus williamsii* Hance is a deciduous shrub or small tree widely distributed in China [1] and used for centuries for the treatment of inflammation [2] and bone fractures and joint diseases [3]. The chemical composition of *S. williamsii* has been extensively studied. Triterpenoids, flavonoids, lignans and the iridoids were reported [4,5]. In our present work, we investigated fraction of the root barks of *S. williamsii* obtained from a macroporous resin by elution with 50% ethanol. Our extraction and separation method can greatly enrich fractions in iridoid compounds so trace iridoids can be isolated. In this paper, we present the isolation and structural characterization of the two new iridoid morronisides (in Figure 1) on the basis of the interpretation of their spectral data, including 1D, 2D NMR and HRESIMS data. 

## 2. Results and Discussion

Compound **1** was obtained as a white amorphous powder and showed positive results for the Molisch reagent. Its molecular formula was determined as C_29_H_46_O_21_ by the positive HRESIMS data. The UV spectrum of the compound displayed an absorption maximum at 239 nm, which is the characteristic of an iridoid skeleton, and intense IR bands at 3,450 and 1,708 cm^−1^, which indicated the presence of hydroxyl and ester carbonyl functionalities, respectively.

Detailed interpretation of 1D and 2D NMR data of **1** confirmed an iridoid structure, the presence of two glucopyranosyl and a fructofuranosyl moieties. The ^1^H-NMR spectrum (Table 1) of **1** in CD_3_OD showed signals diagnostic for an iridoid glycoside at *δ*_H_ 5.89 (1H, d, *J* = 9.2 Hz, H-1), 7.50 (1H, s, H-3), 4.78 (1H, d, *J* = 7.6 Hz, H-1′) and 5.43 (1H, d, *J* = 3.8 Hz, H-1′′′). In addition, the signals at *δ*_H_ 3.68 (3H, s) and 1.33 (3H, d, *J* = 6.8 Hz) were attributed to Me-12 and Me-10, respectively. The ^13^C-NMR spectrum of **1** (Table 1) showed resonances for 29 C-atoms, including two quaternary carbons, six methines, one methylenes, and two methyls belonging to the aglycone moiety, two glucopyranosyl groups and a fructofuranosyl group.

Comparison of the NMR data of **1** and β-morroniside [4] indicated the presence of an additional fructofuranosyl and an additional glucopyranosyl unit. In addition, the NMR data of C-6′ of **1** were significantly deshielded by comparison with those of β-morroniside. This indicated that **1** was a β-morroniside derivative with a fructofuranosyl and a glucopyranosyl moieties located at C-6′, which was verified by correlations from H_2_-6′ to C-4′′, from H-4′′ to C-6′, from H-1′′′ to C-2′′ and from H-2′′ to C-1′′′ in the HMBC spectrum of **1**. Acid hydrolysis of **1** afforded glucose and fructose, which were identified by TLC comparison with authentic samples. The glucose and fructose isolated from the hydrolysate gave optical rotations of [α]_D_^20^ +35.3 (c 0.05, MeOH) and [α]_D_^20^ −90.2 (c 0.025, MeOH), respectively, indicating that they were D-glucose and D-fructose, respectively [6,7,8,9]. The coupling constants of anomeric signals indicated that terminal glucosyl linkage is in α-configuration and inner glucosyl linkage is in β-configuration. Comparison of ^13^C-NMR chemical shifts with literature data [10,11] indicated D-fructose in **1** is in β-configuration.

**Figure 2:**
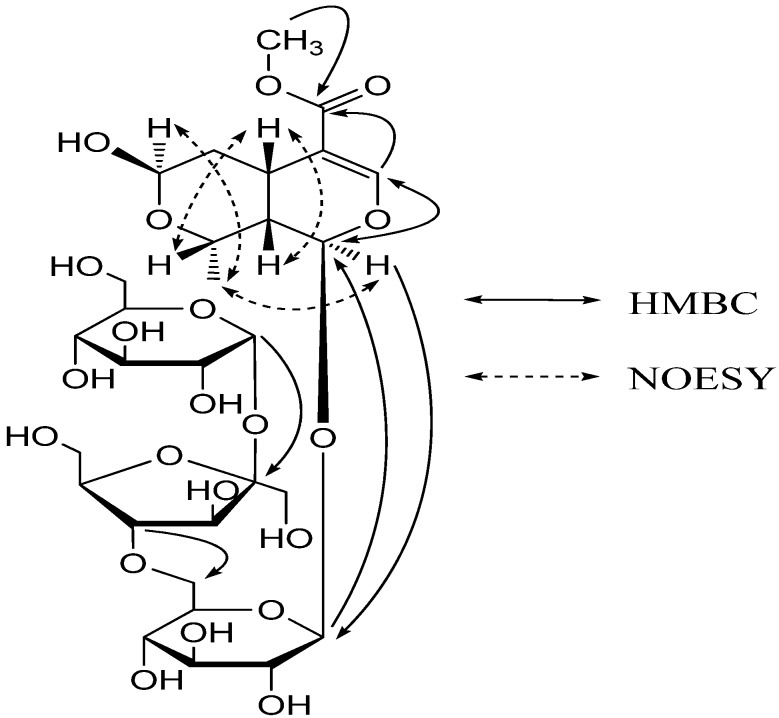
Key HMBC correlations of compound **1**.

The stereochemistry of **1** was established based on the NOESY experiment (Figure 2). The NOESY spectrum showed correlations of H-1/Me-10, Me-10/H-7, H-5/H-8 and H-5/H-9. These data indicated that compound **1** has the same stereochemistry at C-5, C-7, C-8 and C-9 as that of β-morroniside and the hydroxy group bound to C-7 is in the β orientation. Thus, the structure of **1** was identified to be α-D-glucopyranosyl (1→2)-β-D-fructofuranosyl (4→6)-β-morroniside, with the structure shown in Figure 1, and it was named williamsoside C.

Compound **2** was obtained as a white amorphous powder and showed positive results for the Molisch reagent. Its molecular formula was established as C_36_H_54_O_21_ by the positive HRESIMS data. The IR absorption bands at 3,355 and 1,692 cm^−1^, respectively, indicated the presence of hydroxyl groups and a carbonyl group in the molecule.

The ^1^H- and ^13^C-NMR spectra (Table 1) of **2** resemble those of β-morroniside [4], but is more complex. Whereas the ^1^H- and ^13^C-NMR spectrum of **2** shows two sets of signals, this distribution of signals is consistent with a dimeric structure. The analysis of NMR spectra indicated the presence of two distinct iridoid units, which are hereafter referred to as units A and B. The ^1^H-NMR spectrum showed signals at *δ*_H_ 7.50 (1H, s), 5.84 (1H, d, *J* = 9.5 Hz), 1.39 (1H, dt, *J* = 3.7, 13.6 Hz), 1.85 (1H, dd, *J* = 4.7, 13.6 Hz), 4.76 (1H, br.d, *J* = 4.6 Hz) and 1.29 (3H, d, *J* = 6.9 Hz) which were assigned to H-3, H-1 H-6, H-7 and Me-10 of unit A, based on analysis of the HMQC and HMBC spectra. Analysis of the ^13^C-NMR spectra revealed signal values similar to those reported for 7β-*O*-ethyl morroniside [12]. The remaining spectral data revealed a second iridoid unit due to part B of the new iridoid. Signals at *δ*_H_ 7.50 (1H, s), 5.87 (1H, d, *J* = 9.7 Hz), 1.49 (1H, dt, *J* = 3.7, 13.6 Hz), 1.97 (1H, dd, *J* = 4.6, 13.7 Hz), 4.94 (1H, d, *J* = 3.2 Hz) and 1.34 (3H, d, *J* = 6.9 Hz) in the ^1^H-NMR spectrum were assigned to H-3′′, H-1′′, H-6′′, H-7′′ and Me-10′′ of unit B. One significant difference observed for this part was the lack of an ethyoxyl group. The ^13^C-NMR data of this moiety indicated signals identical to those of β-morroniside [4], which was confirmed by 2D NMR data analysis (Table 1).

**Table 1:** ^1^H- and ^13^C-NMR data for compounds **1** and **2** in CD_3_OD (400 MHz for ^1^H and 100 MHz for ^13^C).

No.	1		No.	2	
	*δ*_C_	*δ*_H_ (*J *in Hz)		*δ*_C_	*δ*_H_ (*J *in Hz)
1	95.7	5.89 (1H, d, 9.2)	1	96.0	5.84 (1H, d, 9.5)
3	154.5	7.50 (1H, s)	3	154.5	7.50 (1H, s)
4	111.7	-	4	111.7	-
5	28.0	3.06 (1H, dt, 4.4, 12.8)	5	27.8	3.06 (1H, m)
6	33.7	1.51 (1H, dt, 4.8, 13.6)	6	34.0	1.39 (1H, dt, 3.7, 13.6)
		1.97 (1H, dd, 3.6, 13.6)			1.85 (1H, dd, 4.7, 13.6)
7	92.7	5.00 (1H, br.d, 3.2)	7	97.8	4.76 (1H, br.d, 4.6)
8	66.5	4.36 (1H, dq, 2.0, 6.8)	8	66.0	4.26 (1H, dq, 2.1, 6.9)
9	40.3	1.83 (1H, m)	9	40.4	1.79 (1H, m)
10	19.7	1.33 (3H, d, 6.8)	10	19.7	1.29 (3H, d, 6.9)
11	168.7	-	11	168.6	-
12	51.8	3.68 (3H, s)	12	51.8	3.68 (3H, s)
1′	100.1	4.78 (1H, d, 7.6)	-OCH_2_CH_3_	63.6	3.41 (1H, o)
2′	75.0	3.21 (1H, m)			3.64 (1H, o)
3′	78.0	3.36 (1H, m)	-OCH_2_CH_3_	15.4	1.19 (3H, t, 7.1)
4′	71.6	3.26 (1H, m)	1′	100.4	4.78 (1H, d, 7.9)
5′	78.6	3.06 (1H, dt, 4.4, 12.8)	2′	75.4	3.20 (1H, m)
6′	69.4	3.84 (2H, o)	3′	78.0	3.37 (1H, m)
1′′	63.8	3.62 (2H, s)	4′	71.8	3.34 (1H, m)
2′′	105.6	-	5′	76.8	3.47 (1H, m)
3′′	78.7	4.10 (1H, d, 8.4)	6′	68.0	3.62 (1H, o)
4′′	76.3	3.99 (1H, t, 8.2)			3.96 (1H, dd, 1.8, 12.0)
5′′	82.1	3.90 (1H, o)	1′′	95.7	5.87 (1H, d, 9.7)
6′′	62.3	3.80 (1H, o)	3′′	154.5	7.50 (1H, s)
		3.72 (1H, o)	4′′	111.8	-
1′′′	93.4	5.43 (1H, d, 3.8)	5′′	28.0	3.06 (1H, m)
2′′′	73.3	3.41 (1H, m)	6′′	34.0	1.49 (1H, dt, 3.7, 13.6)
3′′′	74.8	3.68 (1H, m)			1.97 (1H, dd, 4.6, 13.7)
4′′′	71.4	3.34 (1H, m)	7′′	99.3	4.94 (1H, d, 3.2)
5′′′	74.2	3.84 (1H, m)	8′′	66.4	4.30 (1H, dq, 2.1, 7.0)
6′′′	62.8	3.67 (1H, dd, 4.8, 11.8)	9′′	40.3	1.79 (1H, m)
		3.86 (1H, dd, 2.0, 11.8)	10′′	19.8	1.34 (3H, d, 6.9)
			11′′	168.7	-
			12′′	51.8	3.66 (3H, s)
			1′′′	100.1	4.78 (1H, d, 7.9)
			2′′′	75.0	3.20 (1H, m)
			3′′′	78.0	3.37 (1H, m)
			4′′′	71.6	3.28 (1H, m)
			5′′′	78.5	3.28 (1H, m)
			6′′′	62.8	3.63 (1H, o)
					3.86 (1H, dd, 2.0, 11.6)

The absence of the ethyoxyl of unit B, when compared to β-morroniside, and observation of deshielding of C-6′ (*δ*_C_ 68.0), when compared to the 7β-*O*-ethyl morroniside (C-6′, *δ*_C_ 62.8), was the first indication for the attachment of units A and B between C-6′ and C-7′′. The partial structures A and B were reasonably connected to each other by HMBC correlations. The correlations from H_2_-6′ with C-7′′ and from H-7′′ with C-6′ in HMBC strongly indicated the connection between unit A and B through an ether linkage between C-6′ and C-7′′.

The stereo configuration of the substituent group at C-7 and C-7′′ was determined to be the β-orientation on the basis of the obvious NOESY correlations between H-7/Me-10, Me-10/H-1, H-7′′/Me-10′′ and Me-10′′/H-1′′. On the basis of above data, the structure of **2** was identified to be 7β-*O*-ethyl morroniside-(6′-*O*-7′′)-β-morroniside, with the structure shown in Figure 1, and it was named williamsoside D.

## 3. Experimental

### 3.1. General

Optical rotations were measured with a PE-241 digital polarimeter. UV spectra were recorded on a Shimadzu UV-1601 spectrometer. IR spectra were recorded on a Shimadzu FTIR-8400S spectrometer. NMR spectra were recorded on a Bruker DPX 400 NMR instrument (at 400 MHz for ^1^H-NMR and 100 MHz for ^13^C-NMR). Chemical shifts are given as *δ* values with reference to tetramethylsilane (TMS) used as internal standard, and coupling constants are given in Hz. HRESIMS were carried out on Waters Xevo QTOF mass spectrometer. Preparative HPLC (Waters, Delta 600-2487) was performed on a Hypersil-ODS II column (10 μm, 20 × 300 mm, Yilite, Dalian, China).

### 3.2. Plant Material

The root barks of *S. williansii* were collected in August 2008 from the Fangzheng district, Heilongjiang Province, China, and identified by the author Zhen-Yue Wang. A voucher specimen (20080079) has been deposited at Heilongjiang University of Chinese Medicine, Harbin, China.

### 3.3. Extraction and Isolation

The dried root barks (5.0 kg) were extracted with 95% EtOH (2 × 10 L) for 2 h. The EtOH extracts was concentrated under reduced pressure and fractioned on an AB-8 macroporous resin column (8 × 60 cm) with H_2_O, 50% and 95% EtOH-H_2_O to give three fractions (H_2_O fraction, 50% EtOH-H_2_O fraction, 95% EtOH-H_2_O fraction). The 50% EtOH-H_2_O fraction (52.0 g) was repeatedly column chromatographed on silica gel with a gradient of CHCl_3_/MeOH (15:1→1:1) solvents as eluents to afford 10 fractions: Fraction 1–10. Fraction 4 (20 g) continues silica gel chromatography elution with CHCl_3_/MeOH (10:1 to 5:1) to afford a number of sub-fractions A1–A4. Compound **1** (45.5 mg) was obtained by prep. HPLC chromatography of the sub-fraction A2 (3 g) and elution with MeOH/H_2_O (2:5). A3 (7 g) was separated on ODS-A column with MeOH/H_2_O (1:4 to 1:0) as eluent, to produce five sub-fractions (B1–B5). The sub-fraction B3 (2 g) was purified by prep. HPLC with MeOH/H_2_O (3:10) to afford **2** (35.1 mg). 

*Williamsoside C* (**1**): White amorphous powder. [α]_D_^20^ −16.5 (c 0.075, MeOH). UV (MeOH) λmax (log ε) nm: 239 (3.10). IR (KBr): ν = 3,450, 2,500, 1,708, 1,632 cm^−1^. HRESIMS (positive): *m/z* = 731.2619 (calc. for C_29_H_47_O_21_, 731.2610, [M+H]^+^), ^1^H- and ^13^C-NMR: see Table 1.

*Williamsoside D* (**2**): White amorphous powder. [α]_D_^20^ −12.5 (c 0.05, MeOH). UV (MeOH) λmax (log ε) nm: 241 (3.05). IR (KBr): ν = 3,355, 2,450, 1,692, 1,637 cm^−1^. HRESIMS (positive): *m/z* = 823.3230 (calc. for C_36_H_55_O_21_, 823.3236, [M+H]^+^), ^1^H and ^13^C-NMR: see Table 1.

Acid Hydrolysis of **1** and **2**. To a solution of **1** and **2** (each, 15 mg) in MeOH (5 mL) was added 5% H_2_SO_4_ (5 mL) and the mixture was refluxed for 8 h. Each reaction mixture was then neutralized with saturated sodium carbonate and extracted with ethyl acetate (EtOAc, 2 × 10 mL) to give an aqueous fraction containing sugars and an EtOAc fraction containing the aglycone part. The aqueous phase was dried by using a N_2_ stream. The residues were separately subjected to CC over silica gel with MeCN-H_2_O (8:1) as the eluent to yield glucose and fructose from 1, and glucose from 2, respectively. The solvent systems Me_2_CO/H_2_O/CHCl_3_/MeOH (15:1:2:2) and CHCl_3_/MeOH/water (6:4:1) [6,7] were used for TLC identification of glucose and fructose.

## 4. Conclusions

As a part of our chemical investigation on *S. williamsii*, two new iridoid glycosides, α-D-glucopyranosyl (1→2)-β-D-fructofuranosyl (4→6)-β-morroniside (**1**) and 7β-*O*-ethyl morroniside-(6′-*O*-7′′)-β-morroniside (**2**) were isolated. Their structures were established on the basis of spectroscopic evidence. The discovery of compounds **1** and **2** represents a further addition to number and diversity of iridoid glycoside compounds.
